# Epitaxial CdSe/PbSe Heterojunction Growth and MWIR Photovoltaic Detector

**DOI:** 10.3390/ma16051866

**Published:** 2023-02-24

**Authors:** Lance L. McDowell, Milad Rastkar Mirzaei, Zhisheng Shi

**Affiliations:** The School of Electrical and Computer Engineering, University of Oklahoma, Norman, OK 73019, USA

**Keywords:** mid-infrared detector, MBE, heterostructure, lead-chalcogenides, photovoltaic detector, epitaxial growth, RHEED, PbSe, CdSe

## Abstract

A novel Epitaxial Cadmium Selenide (CdSe) on Lead Selenide (PbSe) type-II heterojunction photovoltaic detector has been demonstrated by Molecular Beam Epitaxy (MBE) growth of n-type CdSe on p-type PbSe single crystalline film. The use of Reflection High-Energy Electron Diffraction (RHEED) during the nucleation and growth of CdSe indicates high-quality single-phase cubic CdSe. This is a first-time demonstration of single crystalline and single phase CdSe growth on single crystalline PbSe, to the best of our knowledge. The current–voltage characteristic indicates a p–n junction diode with a rectifying factor over 50 at room temperature. The detector structure is characterized by radiometric measurement. A 30 μm × 30 μm pixel achieved a peak responsivity of 0.06 *A*/*W* and a specific detectivity (D*) of 6.5 × 10^8^ Jones under a zero bias photovoltaic operation. With decreasing temperature, the optical signal increased by almost an order of magnitude as it approached 230 K (with thermoelectric cooling) while maintaining a similar level of noise, achieving a responsivity of 0.441 *A*/*W* and a D* of 4.4 × 10^9^ Jones at 230 K.

## 1. Introduction

Lead selenide has a long history in Mid-Wave Infrared (MWIR) optoelectronic development, with more recent attention focused on the fabrication of MWIR detectors pushing beyond the cryogenic barrier. A room-temperature high-detectivity (D*) MWIR PbSe Photoconductor (PC) has been demonstrated [1,2,3]. To date, low-cost PbSe MWIR photoconductors operating at uncooled or Thermoelectric (TE)-cooled temperatures remain the choice for many sensing and imaging applications [4]. Such high performance is attributed to its low Auger recombination rate at high temperatures. The Auger coefficient in IV-VI semiconductors such as PbSe is roughly an order of magnitude lower than those in Sb-based type-II quantum wells (QWs), which are in turn significantly suppressed relative to other III-V and II-VI semiconductors such as Mercury Cadmium Telluride (MCT) with the same energy gaps [5,6,7,8,9,10,11]. Although very promising performance has been demonstrated by polycrystalline PbSe PC Focal Planar Arrays (FPA), several problems are still associated with their design, including film inhomogeneity, large 1/f noise and limited FPA resolution due to contact configuration for PC detectors [12,13,14,15,16,17,18]. Ideally, if one could develop PbSe photovoltaic detectors with the same performance as their PC counterpart, these problems could be solved. Due to fast dopant diffusion, it is hard to make an abrupt p–n homojunction. Therefore, the heterostructure p–n junction would be one potential option. The challenge for forming a heterojunction is finding a substrate that PbSe can grow on and, at the same time, making a viable type-II heterostructure. We proposed an n-type PbSe/p-type germanium type-II heterostructure [19,20]. Although there is more than an eight percent lattice mismatch between germanium and PbSe, single crystal PbSe was successfully grown on a surface-treated vicinal germanium substrate, resulting in an I-V curve with a very high rectification factor. Despite this, additional work is required to optimize the growth conditions and enhance the detectivity.

II-VI/IV-VI heterostructures such as PbSe/CdSe(S) offer another promising alternative. Initial demonstrations of thermally evaporated polycrystalline CdS thin films on PbSe were fabricated to produce the photovoltaic structure for MWIR detection. While the initial results from these experiments have shown great potential, issues with photogenerated carrier blocking and band alignment issues at lower operating temperatures have stunted their progress [21]. The group II-selenide CdSe and group IV-selenide PbSe form an interesting pair. As shown in Table 1, the lattice constant of CdSe (6.08 A) and PbSe (6.12 A) is close (0.66% mismatch). However, their bandgaps of bulk materials are significantly different, with CdSe being a “wide” bandgap material and PbSe being a narrow bandgap material with room temperature bandgaps of 1.71 eV and 0.27 eV, respectively [22,23,24]. These properties offer opportunities to fabricate nearly lattice-matched high-quality PbSe/CdSe heterogeneous devices. CdSe has been widely used as the shell material for PbSe/CdSe core/shell colloidal quantum dots [25,26,27,28,29,30,31,32,33]. Two types of crystal structures, cubic zincblende and wurtzite hexagonal crystal structures, have been reported for CdSe with different bandgap energies [23,24,34]. Reported band alignments between CdSe and PbSe are not consistent, with the reported electron affinity value of CdSe either slightly higher or lower than that of PbSe, resulting in either type-I or type-II band alignment [35,36,37,38].

In previous works, we have investigated the polycrystalline II-VI n-CdSe films and IV-VI p-PbSe heterojunctions [39,40]. While PbSe has a cubic rock salt crystal structure, polycrystalline CdSe films typically include a hexagonal crystal structure [39]. Such structural differences could introduce interface defects, likely forming a type-I band alignment. In this paper, we report a new method for fabricating an all-epitaxial single-phase (cubic) n-CdSe/p-PbSe heterostructure and link its changes in detector behavior to its polycrystalline II-VI predecessors. The aim of this paper is to fabricate an uncooled photovoltaic mid-infrared photodetector with a decent performance. The growth of a single-phase (cubic-zincblende) epitaxial CdSe thin-film on single crystalline PbSe has never been previously reported, and its demonstration here showcases its potential for the future design of enhanced MWIR PbSe-based photovoltaic detectors. In the following sections, the growth method of the heterostructure is discussed, then the material quality is characterized with RHEED and X-ray diffraction methods. Then, the IV characteristic is shown, and the radiometric measurement is carried out.

## 2. Experimental Methods

### 2.1. Epitaxial Heterojunction Growth

The epitaxial n-CdSe/p-PbSe heterostructure was deposited by MBE on freshly cleaved BaF_2_ (111) substrate with a background pressure of 1 × 10^−8^ Torr. BaF_2_ substrates were used for their excellent long-wave optical transmittance and similar crystal structure with PbSe, enabling epitaxial growth and subsequent back-side illumination detector design for device testing. Then, 1.2 μm PbSe thin films were deposited by direct evaporation of 99.9999% purity PbSe with 0.1% Se-rich effusion source (49.9 wt% lead atoms and 50.1 wt% selenium atoms) in combination with 99.9999% purity elemental Se effusion source to achieve a p-type carrier concentration of 2 × 10^17^ cm^−3^. Epitaxial 200 nm CdSe thin films were then deposited using a 99.999% purity CdSe compound effusion source in combination with a 99.999% purity Bi_2_Se_3_ effusion source to achieve n-type doping.

### 2.2. Epitaxial Heterojunction Growth

A back-side illumination detector structure was fabricated by a photolithography process involving etching of CdSe mesa using a 3:1:1 mixture of 35% HCl acid, 85% phosphoric acid and DI water for 20 sec. Se washing was necessary immediately following the CdSe etching to remove the deposited selenium residue from the sample surface. This process consists of 98 wt% H_2_SO_4_ + 30 wt% H_2_O_2_ in a 3:1 volume-to-volume ratio.

**Figure 1:**
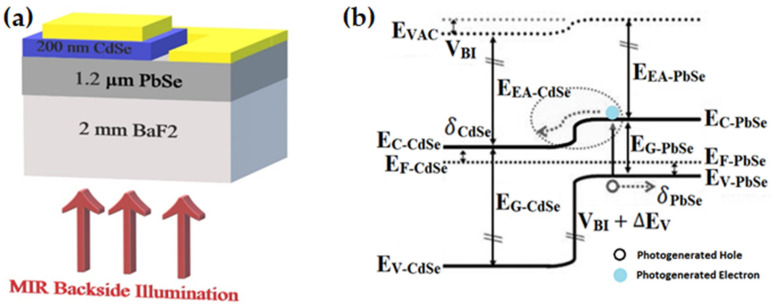
(**a**) Configuration schematic of the n-CdSe/p-PbSe heterojunction mid-IR photodiode with back-side illumination 1.2 μm PbSe deposited on 2 mm BaF_2_ substrate with 200 nm CdSe film deposited on top; 300 nm thick gold deposited on top the CdSe pixel and PbSe to create the contacts. (**b**) suggested energy band diagram of the n-CdSe/p-PbSe heterostructure (in the figure, E _vac_, E_C_, E_F_, E_V_, E_EA_, V_BI_, E_G_, ΔE_V_, δ stands for vacuum energy, conduction band energy, Fermi energy, valence band energy, electron affinity, built-in potential, energy band gap, valence band offset, and energy difference between Fermi energy and conduction band, or Fermi energy between valence band, respectively).

After forming the mesa structure, 300 nm gold was thermally deposited using Lesker Nano 36, and then lift-off of the gold contacts was performed, and the sample was wire bonded using a wedge gold wire bonder for current–voltage analysis and radiometric measurements. The proposed band structure diagram in Figure 1b predicts a type-II heterojunction formation between an n-doped cubic CdSe epitaxial thin film with a p-PbSe layer. After PbSe absorbs the mid-infrared light, an electron–hole pair is generated. Then, the electron–hole pair is separated by the heterostructure’s built-in potential. Finally, the electrons are diffused into CdSe, and holes are transported within the PbSe layer until they reach the contact.

## 3. Results and Discussion

### 3.1. Crystallography and Surface Morphology of Epitaxial CdSe Films on PbSe

RHEED is an in situ powerful tool in MBE to monitor and evaluate the real-time growth procedure. RHEED employs an electron gun to graze the substrate with high-energy electrons. Based on the atomic spacing and crystal structure of the surface of the sample, as well as the wavelength of the incident electrons, diffraction results in the constructive interference of the electrons at specific angles. We used this powerful tool to watch the layer-by-layer growth. In situ RHEED monitoring of the growth evolution was captured and analyzed using the KSA 400 data acquisition and analysis software tool. X-ray diffraction (XRD) was used to corroborate the RHEED observations and examine the surface morphology and crystallography of the epitaxial CdSe thin films on PbSe. In situ RHEED measurements were performed during the MBE deposition of lead selenide and cadmium selenide epitaxial films on barium fluoride substrates. Special interest was focused on the nucleation and growth evolution of CdSe thin films on PbSe since the single phase (cubic) epitaxial growth of CdSe on the IV-VI rock salt crystal structure has never been reported.

**Figure 2:**
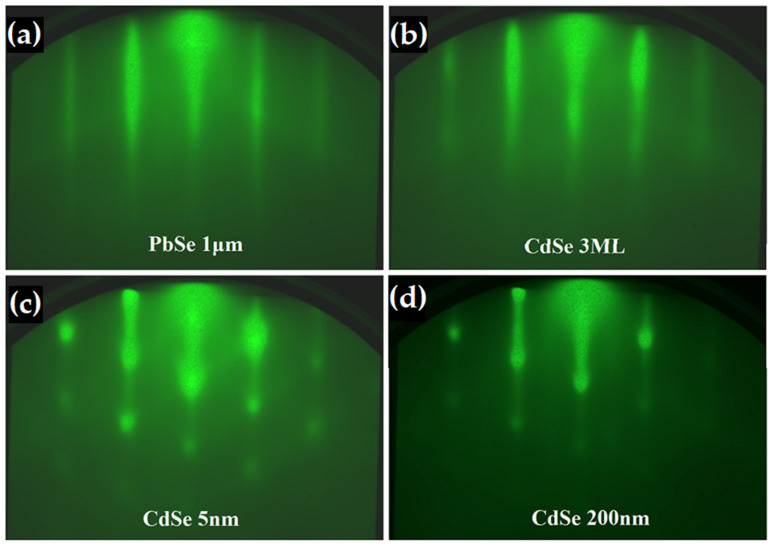
In situ RHEED measurements of the characteristic [110] orientation for (**a**) 1 μm thick PbSe film on BaF_2_, (**b**) 3 ML nucleation of CdSe on PbSe, (**c**) 5 nm of CdSe, and (**d**) final 200 nm bulk CdSe thin film. The nucleation on growth of CdSe suggest that the CdSe followed the PbSe crystal structure and orientation.

Observing Figure 2, the nucleation of CdSe shows a strong correlation with the growth surface, matching the structure and orientation of the PbSe seed layer while proceeding in a layer-by-layer growth mode. After the initial deposition accumulation exceeds 5 nm, RHEED observations begin to show signs of roughening, with the formation of 3D spots along the <110> direction. The rest of the bulk 200 nm thick CdSe growth proceeds virtually unchanged, with no noticeable contributions from varying CdSe crystallites, such as off-orientation cubic CdSe grains or the formation of a secondary phase of wurtzite CdSe. Interestingly, line profile analysis of the CdSe RHEED images shows an identical diffraction spacing between the <110> CdSe and the <110> PbSe fringes. This indicates that the epitaxial growth of CdSe maintained roughly the same lattice parameter as PbSe during the bulk of the 200 nm thin film growth. Only during the last few nanometers of thickness did the line spacing begin to increase, indicating a decrease in the lattice parameter. While PbSe has a relaxed lattice constant of 6.12 Å, the literature indicates an expected lattice constant of 6.08 Å for cubic CdSe. However, here we observe a slightly larger lattice parameter for the bulk CdSe film deposition closer to the 6.12 Å of PbSe. This phenomenon is further investigated in the following XRD measurement and analysis of the as-grown heterostructure.

**Figure 3:**
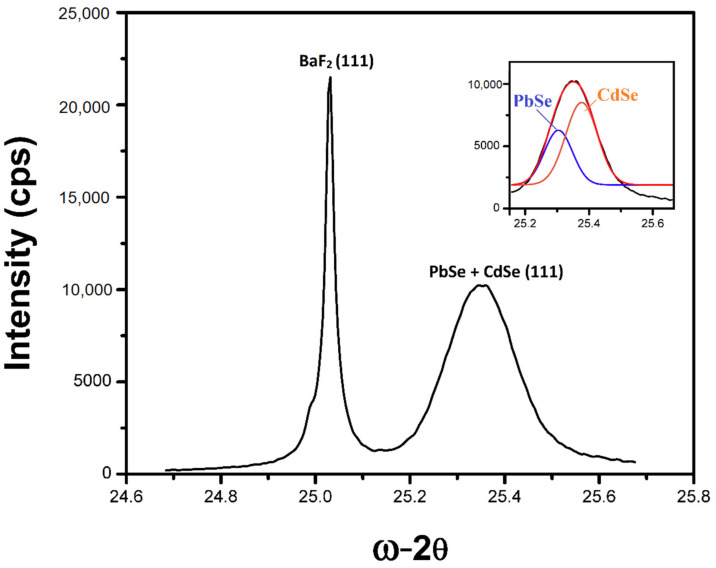
XRD measurement of the epitaxial n-CdSe/p-PbSe heterostructure on BaF_2_ (111) substrate. The inset shows a simulated overlapping peak of PbSe and CdSe.

High-resolution X-ray diffraction measurements were taken of the as-grown CdSe/PbSe heterostructure on BaF_2_. XRD scans of the main (111) substrate and layer peaks for PbSe and CdSe show a striking overlap of their diffraction peak contributions, as was expected from the RHEED investigation. The inset image in Figure 3 shows the deconvoluted peak contributions from the PbSe and CdSe layers, with a PbSe (111) peak position of 25.30° and a CdSe peak position of 25.38°. No off-orientation peak contributions were observed for either PbSe or CdSe, and neither were there any contributions from wurtzite CdSe, with characteristic peak positions at 23.4°, 24.8°, 26.5°, and 38.4°. The observations here corroborate the in situ RHEED measurements observed during the growth of CdSe on PbSe, indicating unambiguously the successful formation of a single phase (cubic) bulk epitaxial CdSe thin film deposited directly on PbSe.

### 3.2. Current-Voltage and Radiometric Measurements

Processing of a 30 μm × 30 μm pixel for I-V measurement was performed to investigate the potential p–n junction behavior between the epitaxial n-CdSe and p-PbSe films. Current-voltage measurements were performed using a Keithley 2400 source meter instrument.

**Figure 4:**
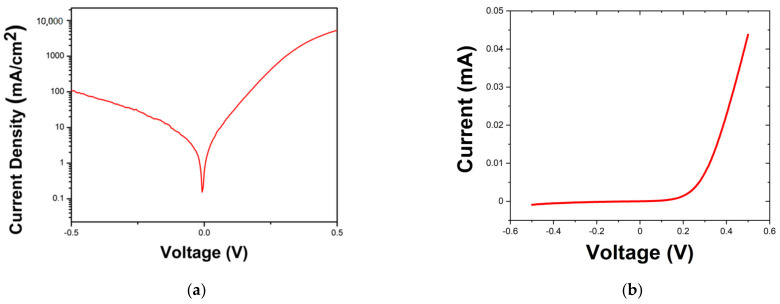
(**a**) Room-temperature J-V and (**b**) I-V characteristic of the epitaxial n-CdSe/p-PbSe heterojunction structure shows a strong room temperature p–n junction behavior.

Figure 4a shows the J-V statistics for the as-grown n-CdSe/p-PbSe heterojunction structure, demonstrating strong room-temperature p–n junction behavior similar to previous investigations on poly-CdSe/PbSe devices [40]. Notably, a strong rectifying factor is observed, indicating the formation of a barrier for the reverse bias current, with a rectifying factor of 50 when comparing the current density at 0.5 V forward bias and reverse bias, respectively (Figure 4b). Subsequently, the reverse bias current density at −100 mV is only 7.5 mA/cm^2^. While reports of similar levels of dark current density have been observed before in PbSe-based PV structures, the report here distinguishes itself in that the low leakage current was observed for the as-grown material structure without any post-growth passivation treatments.

**Figure 5:**
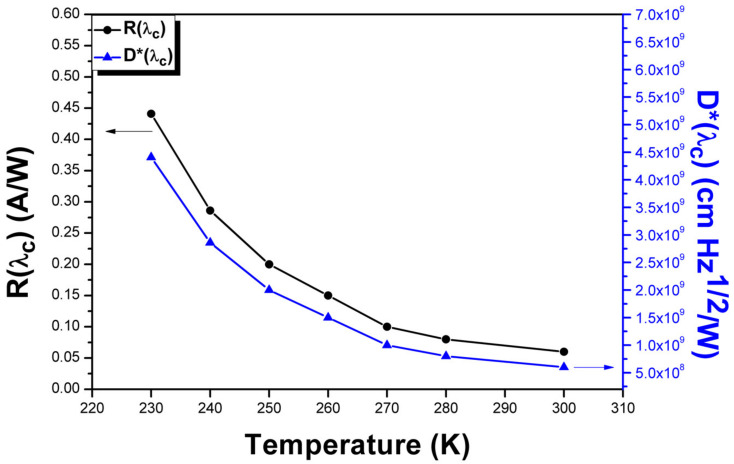
Temperature-dependent peak responsivity (black curve) and D* (blue curve) measurements from 300 K down to 230 K under zero bias photovoltaic mode. It shows almost an order of magnitude of higher signal when the device is cooled down to 230 K.

Figure 5 shows temperature-dependent radiometric measurements, which were performed from room temperature down to 230 K to investigate the signal-dampening phenomenon observed in our previous CdSe/PbSe efforts. The as-grown n-CdSe/p-PbSe structure displays a temperature-dependent signal increase at lower temperatures while maintaining similar noise levels resulting in higher detectivities.

To measure the specific detectivity and responsivity, a calibrated 500 °C blackbody from Infrared System Development was used as the infrared light source. A Thorlabs mechanical chopper was used to modulate the mid-infrared light of the blackbody with a 750 Hz chopping frequency. Measurements were conducted under the zero-bias condition. The signal and noise currents from the device are collected from a Stanford Research System SR830 lock-in amplifier. The Responsivity (*R*) and Specific Detectivity (D*) were calculated using the following formulas.

(1) $$D^{*}=R*\frac{\sqrt{A*\Delta f}}{I_{n}}\left(Jones\right)$$

(2) $$R=\frac{I_{s}}{P_{i}}\left(A/W\right)$$

where *I_s_* and *I_n_* are the measured detector output signal and noise currents, *A* is the device detection area, Δf is the noise bandwidth, and *P_i_* is the incident radiant power. The room temperature 30 μm × 30 μm pixel achieved a peak responsivity of 0.06 *A*/*W* and a D* of 6.5 × 10^8^ *Jones* under the zero bias photovoltaic operation. With decreasing temperature, the optical signal increased by almost an order of magnitude as it approached 230 K while maintaining a similar level of noise, achieving a responsivity of 0.441 *A*/*W* and a D* of 4.4 × 10^9^ *Jones* at 230 K. The temperature-dependent behavior observed in this new single-phase epitaxial n-CdSe/p-PbSe heterojunction device sheds light on the inverted temperature-dependent behavior phenomenon observed by the previous explorations of similar CdSe/PbSe PV detector structures. Here, this new epitaxial interface between cubic PbSe and CdSe films without any contributions from hexagonal-CdSe grains demonstrates the first example of the CdSe/PbSe PV detector structure’s continued increase in signal and detector performance down to thermoelectric-cooled operating temperatures. Further, the as-grown detector performance of this new technology demonstrates an almost 3 × D* increase at room temperature compared to the as-grown poly-CdSe/PbSe PV detector structure. Combined with the previous studies on device enhancement using post-growth treatment techniques and enhanced device structure design, this new epitaxial p–n heterojunction structure demonstrates a promising advancement in the fabrication of enhanced II-VI/IV-VI interfaces and heterojunctions, with the potential for developing new state-of-the-art MWIR photodetectors operating above cryogenic cooling temperatures.

## 4. Conclusions

This study exhibits the integration of epitaxial n-CdSe films on p-PbSe films on BaF_2_ substrates, resulting in a novel p–n heterogeneous structure with outstanding material quality, promising J-V statistics, and decent high-temperature detectivity. In situ and ex situ characterization of the resulting heterogeneous structure shows the formation of a high-material-quality epitaxial single-orientation CdSe on PbSe. Future work will focus on further improving and understanding the growth mechanism and growth of the material system on CaF_2_-Silicon substrate to utilize the scalability of the technology to the silicon technology standard. Further extending the PbSe absorption spectra via ternary Pb_1−x_Sn_x_Se compound films into the long-wave infrared (LWIR) regime and integrating the n-CdSe/p-PbSe heterostructure on silicon may push the limits of this material structure and open doors to new and intriguing technological fronts.

## Figures and Tables

**Table 1:** Material properties of PbSe and CdSe.

Material	Crystal Structure	Lattice Constant (Å)	Bandgap (eV)	Reference
PbSe	Rock Salt	6.12	0.27	[22]
CdSe	Zincblende	6.08	1.71	[23,24]
CdSe	Hexagonal	4.3	1.8	[24,34]

## Data Availability

Data are available through email upon a reasonable request.
